# The Upside to Feeling Worse Than Average (WTA): A Conceptual Framework to Understand When, How, and for Whom WTA Beliefs Have Long-Term Benefits

**DOI:** 10.3389/fpsyg.2020.00642

**Published:** 2020-04-08

**Authors:** Ashley V. Whillans, Alexander H. Jordan, Frances S. Chen

**Affiliations:** ^1^Department of Negotiations Organizations and Markets, Harvard Business School, Harvard University, Cambridge, MA, United States; ^2^Harvard Medical School, Cambridge, MA, United States; ^3^Department of Psychology, Vancouver, BC, Canada

**Keywords:** social comparisons, worse than average, better than average, social cognition, self-perception

## Abstract

Our thoughts, feelings, and behaviors are shaped in critical ways by our beliefs about how we compare to other people. Prior research has predominately focused on the consequences of believing oneself to be *better* than average (BTA). Research on the consequences of worse-than-average (WTA) beliefs has been far more limited, focusing mostly on the downsides of WTA beliefs. In this paper, we argue for the systematic investigation of the possible long-term benefits of WTA beliefs in domains including motivation, task performance, and subjective well-being. We develop a conceptual framework for examining these possible benefits, we explore the usefulness of this framework to generate novel insights in an important psychological domain (skill learning), and we conclude with broader recommendations for research in other domains such as friendship formation, moral, and political decision making.

## Introduction

On the day before your annual performance review, you might have one of two thoughts: you might think that you are *less* skilled than your fellow colleagues or you might think you are *more* skilled. If you are like most people, your thoughts will likely align with the latter option – you will confidently believe that you are more skilled than your peers. Most people believe that they are “better than average” (BTA): more intelligent, interesting, and attractive than other people (Alicke et al., 1995). As it turns out, BTA beliefs are linked to short-term psychological benefits such as positive mood and enhanced self-esteem (Aspinwall and Taylor, 1993). However, what if you believed that you were less skilled than your peers? You might initially feel bad about yourself, but could there also be hidden upsides to feeling “worse than average” (WTA) – such as superior skill learning and long-term professional advancement?

Prior research has predominately documented the downstream consequences of BTA beliefs for motivation, task performance, and subjective well-being (Wills, 1981; Taylor and Brown, 1988; Robins and Beer, 2001). Researchers have provided a balanced account that includes both the positive and negative consequences of BTA beliefs for task performance, well-being, and social connection (e.g., Leventhal, 1976; Moore and Kim, 2003; Gino and Moore, 2007). In contrast to this measured research on BTA beliefs, much less research has focused on WTA beliefs. And, most of the research that has been conducted on WTA beliefs has primarily focused on the negative consequences.

The potential positive consequences of WTA beliefs may have been overlooked in part because WTA beliefs stand in stark opposition to strong cultural ideals in North America. The current zeitgeist in North American culture promotes self-enhancement and high self-regard (Twenge and Campbell, 2010) and scientists are not exempt from culturally-biased thinking (Henrich et al., 2010). In fact, researchers have made omissions about other less culturally-desirable traits. The benefits of introversion and solitude are only recently being systematically documented after delayed investigation (Kahnweiler, 2009; Grant, 2013), and there is a recent upsurge of research examining the benefits of negative affective experiences such as depressed mood (Andrews and Thomson, 2009; Kashdan and Biswas-Diener, 2014) and conversely, the downsides of positive affective experiences such as happiness (Gruber et al., 2011; Mauss et al., 2012).

We propose that a systematic attempt to document the benefits of WTA beliefs is long overdue; thus, our overarching aim is to encourage more research on this potentially rich topic in social cognition. To this end, the current paper develops a conceptual framework to theorize about when and for whom WTA beliefs are likely to have positive downstream consequences for motivation, task performance, and well-being. In contrast to previous theoretical models, which have focused primarily on the causes (Chambers and Windschitl, 2004; Moore and Small, 2007; Guenther and Alicke, 2010) or immediate consequences of social comparison processes (Tesser et al., 1988; Aspinwall and Taylor, 1993), our conceptual framework maps out a sequence of affective and cognitive events that could allow the benefits of WTA beliefs to accrue over time. We also specify how individual differences influence the progression of this sequence. To demonstrate the relevance of this framework, we apply it to the example domain of skill learning, which is a critical determinant of task performance as well as subjective well-being (Reis et al., 2000; Diener and Seligman, 2002). To conclude, we speculate about the usefulness of this framework for other psychological domains ranging from friendship formation to moral and political psychology.

Prior research has pointed to the role of stable personal characteristics in predicting motivation and action tendencies in response to negative feedback and perceived threat – including optimism (Carver and Scheier, 2001), incremental theories (Dweck, 2007), self-efficacy (Deci and Ryan, 2010), and consistent positive role models (Lockwood and Kunda, 1997). Extending this foundational research, we explore a previously-overlooked situational factor. Specifically, we explore how the extent to which the current context incites WTA beliefs predicts motivational tendencies and behavioral remediation. Thus, this paper builds on foundational theories of human motivation to understand the unique role of WTA beliefs in predicting positive long-term changes in motivation and behavior.

### WTA/BTA Beliefs in Relation to Other Self-Evaluations

BTA beliefs occur when people think that their standing on some dimension (e.g., a skill, a trait, or their chance of success) is superior to that of the average person or peer. In contrast, WTA beliefs occur when people think that their standing on some dimension is inferior to that of the average person or peer. WTA and BTA beliefs are conceptually related to underconfidence and overconfidence (i.e., when people are unrealistically pessimistic/optimistic about their chance of experiencing positive events (Weinstein, 1980) as well as to self-effacement and self-enhancement (i.e., when people demonstrate a preference to hold unrealistically negative/positive beliefs about themselves; Brown, 1986; Taylor and Brown, 1988, 1994; Colvin and Block, 1994). Given that there is limited research exploring the long-term consequences of BTA and WTA beliefs, we will also review research that is relevant to these and related constructs. However, we observe two important distinctions between WTA beliefs and underconfidence/self-effacement. Underconfidence and self-effacement are predicated on beliefs about the self: believing that you are or are not performing according to your own standards or believing that you are or are not likely to experience certain events. In contrast, WTA beliefs involve a salient social comparison: believing that you are worse than or better than the average person or peer. Thus, we propose that WTA beliefs are particularly likely to trigger *socially-oriented* affective, cognitive, and behavioral outcomes. That is, we propose that WTA beliefs are likely to lead to psychological and behavioral outcomes that rely on seeking out relevant social models or social feedback (Seta, 1982). We also propose that the feeling of not performing as well as one’s peers – as opposed to simply feeling dissatisfied with one’s performance or abilities – is uniquely motivating (Shore and Tashchian, 2002). We will further expand and contextualize these arguments in the conceptual framework detailed below.

## Immediate Consequences of BTA and WTA Beliefs

BTA beliefs are very common: research suggests that individuals typically see themselves as better than their peers on personal characteristics ranging from physical attractiveness to leadership abilities (Taylor and Armor, 1996). The immediate consequences of BTA beliefs include boosts in momentary affect and subjective well-being (Gibbons and Gerrard, 1989; Testa and Major, 1990; Aspinwall and Taylor, 1993; Major et al., 1993) as well as gains in task performance (Ehrlinger and Dunning, 2003). In contrast, WTA beliefs have been linked to negative momentary affect and decrements to subsequent task performance. For example, participants who received feedback that they had performed worse than one of their peers on a personally-relevant task experienced more arousal and greater negative affect compared to participants who received feedback that they had performed better or equally as one of their peers. These negative affective responses predicted poorer performance on subsequent lab tasks, due to behaviors such as speeding up while completing tasks that required focus and careful attention (Tesser et al., 1988).

In another set of studies, students who believed that they were unskilled at a task also believed that they were taking more time to answer questions and were expending more effort on the task compared to students who believed that they were skilled at the domain in question, regardless of their actual performance (Critcher and Dunning, 2009). Skill-based misperceptions have negative immediate consequences for task performance: for example, negative skill-based misperceptions are associated with reduced performance on spatial, numerical, and verbal tasks (Paunonen and Hong, 2010), poorer public speaking performance (Gilovich and Savitsky, 1999), and worse performance on novel tasks in the lab (Zunick et al., 2015). Overall, when people are put “on the spot” to perform in a domain where they feel WTA, their performance suffers. In a cross-sectional study, people who overestimated the extent to which their peers experienced positive emotions in comparison to themselves reported lower well-being, greater rumination, and more depressive symptoms (Jordan et al., 2011a). These findings provide evidence that WTA beliefs have negative consequences for momentary affect, immediate task performance, and well-being. However, the reliance on cross-sectional designs and lab-based tasks to draw conclusions about the effects of BTA and WTA-related beliefs may be short-sighted. We suggest that longitudinal designs may reveal a markedly different picture of how these beliefs impact thoughts, feelings and behaviors over a different time scale than has typically been considered in research.

## Consequences of BTA and WTA Beliefs Over Time

Why might the relative balance of benefits and drawbacks of BTA and WTA beliefs change over time? Researchers have speculated that self-enhancement related beliefs result in reduced motivation and efforts to improve (Moore and Healy, 2008; Brown, 2012). Cross-sectional data provides evidence that when students are surrounded by other students with lower academic abilities, they experience greater academic self-esteem, yet show worse academic achievement compared to students who are surrounded by others with higher academic abilities (Marsh and Parker, 1984; Altermatt and Pomerantz, 2005).

Researchers have also speculated that holding overly positive beliefs about one’s abilities can lead to unrealistic expectations and have detrimental consequences for performance by increasing the likelihood of experiencing frustration and burn-out (Polivy and Herman, 2000). In a qualitative study of teachers recruited from workshops in the US and Israel, teachers who reported a greater discrepancy between idealized expectations of their own performance and actual performance reported greater burn-out and less job satisfaction (Friedman, 2000). Building from this cross-sectional evidence, we propose that the benefits of WTA beliefs have been overlooked in part because they tend to unfold over longer timescales than the benefits of BTA beliefs. Indeed, “sleeper” effects have been documented in other domains, such as in the context of clinical treatments for alcoholism (White et al., 2007) and schizophrenia (Moritz et al., 2014): the treatments that are most difficult for patients to experience and adhere to in the short-term often yield the greatest long-term benefits.

Although direct evidence documenting the longer-term consequences of BTA and WTA beliefs is limited, related research suggests that the short-term consequences of self-enhancement and overconfidence in the domains of academics, well-being, and social activities may come at a long-term cost. In one of the few longitudinal studies in this area, college students who overestimated their abilities felt more disengaged and had lower self-esteem and subjective wellbeing 4 years later; students who did not initially overestimate their academic abilities did not show this pattern of decline (Robins and Beer, 2001). In another longitudinal study, students entering college with overly high expectations about their academic achievement reported greater self-esteem at baseline yet showed decreases in self-esteem during their 4-year college degrees, even after controlling for the grades they received (Chung et al., 2014). Overly high expectations might have detrimental consequences over time because people cannot live up to their own expectations, thus providing evidence that having overly positive beliefs can be a “mixed blessing” (Chung et al., 2014).

Detrimental effects of BTA-related beliefs have also been documented for well-being, physical health, and social relationships. Individuals who scored higher on self-enhancement measures reported greater positive affect and resilience up to several months after being personally involved in a traumatic event (September 11th), however, these individuals were also rated by friends and relatives as less socially adjusted 18 months later (Bonanno et al., 2005). College students who were unrealistically optimistic about how alcohol consumption would impact their lives (i.e., students who reported that would have fewer problems with alcohol use compared to peers) showed increases in negative alcohol-related incidents over a 2-year period (Dillard et al., 2009).

In the social domain, people who overestimate how popular and well-liked they are (as compared to how popular peers rate them) are initially liked better, yet, over time, self-effacers are liked more by others (Paulhus, 1998; Anderson et al., 2006). Students who initially engaged in more status self-enhancement during face-to-face group interactions were liked less over four separate interactions compared to students who were initially accurate about their status or were self-effacing; furthermore, groups with a higher number of status self-enhancers experienced more conflict during an in-lab task (Anderson et al., 2006). Research suggests that individuals holding overly positive beliefs about themselves (compared to ratings made by trained examiners and peers) are liked less by others over time (Colvin et al., 1995), go on to receive lower scores on annual performance reviews (Lönnqvist et al., 2008), and experience decreased satisfaction over the course of their romantic relationships (McNulty et al., 2008) and when making the transition to parenthood (Ungerer et al., 1997).

In summary, a number of studies suggest that BTA-related beliefs can incur long-term psychological costs. This work also raises the question of whether WTA beliefs might also incur long-term psychological benefits. Much remains unknown about the time frame and sequence of affective and cognitive events through which WTA beliefs reliably promote positive psychological outcomes. Thus, to guide future research in this area, we propose a conceptual framework that generates predictions about when, how, and for whom WTA beliefs might have long-term benefits for motivation, task-performance, and well-being.

## Conceptual Framework

**FIGURE 1:**
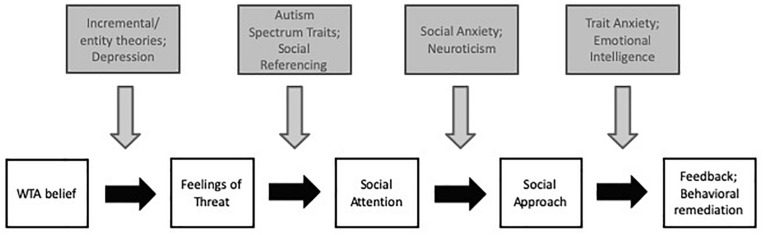
Conceptual framework for a sequence of events that leads to long-term benefits of WTA beliefs. White boxes represent the sequence. Gray boxes represent individual difference factors that can influence how effectively progress is made between steps of the sequence.

We propose that under certain conditions, WTA beliefs trigger a temporally predictable sequence of affective, motivational and behavioral changes events that can promote successful long-term behavioral change (Figure 1). Specifically, we propose that WTA beliefs produce long-term positive consequences when they lead to feelings of threat, enhance attention toward appropriate social models, encourage social approach, facilitate social feedback, and lead to improved motivation and task performance. In our model, we do not focus on why people have WTA beliefs or how accurate these beliefs are, given that this theorizing exists elsewhere. For relevant reviews see Larrick et al. (2007), Benoît et al. (2015). Instead, we focus our conceptual model on looking at what happens after WTA beliefs arise. Regardless of whether or not the WTA belief is accurate, WTA beliefs are likely to trigger a cascade of behavioral and motivational consequences – and under certain circumstances – have positive consequences.

### Feelings of Threat

Consistent with past theorizing (Roese and Olson, 2007), we posit that there are two initial and necessary conditions for WTA beliefs to yield benefits over time: (1) an individual must feel threatened by, and motivated to reduce, a WTA belief and (2) an individual must feel that his or her own standing in the situation is subject to change. Stated differently, successful “behavioral remediation” – actions taken by people toward improving their situations – depends on choosing to reduce the discrepancy between oneself and others and believing that one can (Roese and Olson, 2007). WTA beliefs signal that one’s performance is not adequate, and they are generally perceived as threatening (Taylor and Brown, 1988). In turn, feelings of threat are aversive (Greenwald, 1980; Steele, 1988; Tesser et al., 1988; Wilson et al., 2003), and feelings of threat encourage people to change their behavior (Solomon et al., 1991; Heine et al., 2006; Roese and Olson, 2007).

We propose that WTA beliefs may uniquely motivate behavioral remediation because of a specific desire to feel at least average compared to one’s peers. Put differently, people may be more likely to pursue behavioral remediation to get from the 40th to 50th percentile, than they would be to get from the 51st to 61st percentile. Indeed, Festinger’s (1954) pioneering theoretical work on social comparison processes postulated that social comparisons lead to a motivation to reduce the discrepancy between oneself and others, beyond more general attempts to simply feel better about one’s performance (Hypothesis 1, p. 118). Individuals at the 50th percentile may, in essence, serve as an abstracted average “other” to whom one compares oneself. Consistent with this notion, people experience the greatest motivation to improve after receiving feedback that they are performing worse than average as compared to receiving positive or negative feedback about performance in the absence of normative information (Shore and Tashchian, 2002). Of course, the specific extent to which people are motivated by the average vs. another target could be driven by other factors such as how personally committed someone is to the task. This motivation is also likely to be driven by whether performance is framed as getting closer to a collective goal – in which case, people might be more motivated to perform *better* than their peers versus at the average (e.g., Koo and Fishbach, 2010).

Other individual differences are also likely to play a role in predicting how people respond to threatening situations. For example, personal characteristics such as trait levels of optimism and pessimism (Carver and Scheier, 2001) could also impact whether individuals view certain situations as a threat or as an opportunity. Consistent with prior research (Tesser et al., 1988), WTA beliefs also might not motivate behavior change when (1) people are not personally invested in the domain where WTA beliefs arise and when (2) people do not feel close to or do not identify with the group to which they are comparing themselves. In addition to low group identification, high group identification might also prevent WTA beliefs from triggering the processes necessary for positive behavioral remediation. For example, people who highly identify with a group that they are part of, often start to see others’ success as their own (Cialdini et al., 1976). Thus, if a person’s group is successful, they feel highly identified with this group, and the group accepts them, they might be unlikely to change their behavior because they see their group’s success as their own.

Similarly, different types of threat that are elicited by WTA beliefs could play a role in whether these beliefs have positive or negative or negative consequences. For example, if someone experiences WTA beliefs in a *specific* domain that is less central to their self-worth, they might be less likely to experience anxiety, and more likely to engage in positive behavioral remediation. Yet, if someone experiences WTA beliefs as a *general* threat or in relation to a domain that is central to their self-worth, they might be more likely to experience anxiety and avoid remedying the WTA belief (i.e., engage in avoidance; see Jonas et al., 2014 for a recent theoretical review).

It is also possible that different types of negative emotions could trigger different motivational processes. For example, if people feel shame in response to WTA beliefs, they might disengage from the activity that caused their WTA beliefs. In contrast, if people feel envy or guilt in response to WTA beliefs, they might report greater engagement with the activity and increased improvement in the domain over time (see Schmader and Lickel, 2006). More research is needed to unpack the role of specific negative emotions in predicting the proposed motivational and behavioral cascade.

Overall, our proposed conceptual model reflects a general pattern of motivational and behavioral responses to WTA beliefs. Numerous boundary conditions such as group identification, social acceptance, and others not mentioned here, will no doubt play a role in the extent to which WTA beliefs result in positive long-term remediation. Future research will be needed to explore the relative importance of these and other conceptually-related moderators.

### Social Attention

In the context of WTA beliefs, feelings of threat that arise from inadequate performance are related to how a person is performing in comparison to his or her peer group. Consequently, when feelings of threat are combined with WTA beliefs, a unique situation arises wherein other people are both the cause of (via negative social comparisons), and a potential solution to (as potential models), an individual’s negative affective state. Therefore, we hypothesize that feelings of threat that arise from WTA beliefs are unique, relative to related beliefs such as underconfidence and self-effacement, in their tendency to motivate individuals to focus on others in their social environment.

This step of the conceptual framework posits that successful behavioral remediation that follows from feelings of threat hinges on an individual’s attention being selectively refocused on social models. While a general negative evaluation of oneself or one’s own standing (e.g., underconfidence, self-effacement) could lead to successful behavioral remediation through either social or non-social methods, we propose that WTA beliefs may more directly motivate people to seek out relevant social models^1^. Here, our theorizing is consistent with research suggesting that people are more likely to evaluate their abilities and opinions by comparing their own performance with the abilities and opinions of others, in the absence of objective performance standards (Festinger, 1954). When individuals feel like they are not living up to social standards, they become more motivated to compare themselves with others to learn how to modify their behavior (Gibbons and Buunk, 1999). When people feel uncertain about their performance in a personally-relevant domain, they spend more time comparing themselves to others (Butzer and Kuiper, 2006), such as by spending more time comparing themselves to other people on Facebook (Lee, 2014).

However, for social approach behavior to have adaptive consequences, the social model who is sought out must be appropriate for improving one’s own capacities in a specific domain. Empirical evidence suggests that most people are quite effective at seeking out relevant social models to promote learning. Children who are as young as 5 years old seek out accurate (as opposed to simply confident) models in new domains (Brosseau-Liard et al., 2014). This ability to seek out relevant and appropriate social models may have evolved to facilitate skill learning (Boyd et al., 2011; Chudek et al., 2012). Consequently, to the extent that WTA beliefs lead to feelings of threat and heightened social attention, people are likely to seek out relevant social models to learn from and reduce WTA beliefs.

### Social Approach

Next, our conceptual framework posits that for most people, enhanced attentional focus on others – resulting from WTA-belief-induced feelings of threat – should lead to social approach. Consistent with this possibility, among psychologically healthy individuals, social threats such as negative social evaluation or social rejection increases social approach motivation (Maner et al., 2007; DeWall et al., 2009), prosocial decision-making (Von Dawans et al., 2012), and feelings of closeness with strangers (Berger et al., 2016). The negative affect generated by social threat also leads people to seek out others’ advice (de Hooge et al., 2014). Individuals led to feel anxious were more likely to seek out and take advice that was provided (Gino et al., 2012). We propose that WTA beliefs promote positive long-term behavior changes when people increase their attention to social models, as well as when people socially approach those models. The extent to people approach, seek out, or take the advice of others after experiencing WTA beliefs is likely to depend on the extent to which other people trust those in their social environment (e.g., Balliet et al., 2014). See section “Social Approach: The Role of Social Anxiety and Neuroticism” for a further discussion of this point.

Indeed, various moderators will predict whether perceived social threats translate into social approach versus avoidance. As we will discuss later on in our framework, people who experience high fear of negative evaluation might not respond as positively to the perception of social threats (Maner et al., 2007). Furthermore, people who feel undervalued by the relevant social group might also be less likely to reach out and form social connections with other people from that same group (Maner et al., 2007). Future research is needed to further explore these and related boundary conditions.

### Feedback

Finally, for the benefits of WTA beliefs to accrue over time, individuals need to continuously monitor and modify their behavior in response to social feedback. This proposition is consistent with research suggesting that goal achievement is a dynamic process (Van Yperen and Renkema, 2008). Within our framework, WTA beliefs should dissipate over time as one’s actual or perceived performance improves, and reappear when one’s performance declines (Carver, 2006; Sedikides and Hepper, 2009). Consequently, several iterations of this hypothesized sequence of events – in which WTA beliefs trigger feelings of threat, enhance social attention, and promote social approach and social feedback, may occur before the maximal benefits of WTA beliefs are realized. The time course over which the benefits of WTA beliefs unfold will vary depending on domain-specific factors, such as how long it takes to learn a skill as well as the level of proficiency an individual hopes to achieve (Carver, 1978). This dynamism speaks to the importance of future research documenting long-term consequences of WTA beliefs.

## Individual Differences

Thus far, our model has provided an overview of a specific sequence of events through which WTA beliefs should lead to positive consequences: namely, when people feel threatened, turn to social stimuli, learn from relevant social models, and update their behavior in response to social feedback. Yet, our framework also posits that WTA beliefs are not uniformly beneficial; we predict that individual differences influence who will engage in the affective processes, cognitions and behaviors that are likely to promote the positive long-term consequences of WTA beliefs. Although the following list is not intended to be exhaustive, the overarching idea is that these specific individual differences will affect people’s reactions at each junction of the model, thereby determining whether and how the benefits of WTA beliefs accrue over time (Figure 1).

### Feelings of Threat: The Role of Entity/Incremental Theories and Depression

Individual differences related to how people respond to threatening situations likely play a crucial role in predicting the long-term benefits of WTA beliefs. First, people differ in their belief that various personal characteristics, from intelligence to athletic prowess, are fixed and trait-like (entity theory) or malleable and changeable via effort and hard work (incremental theory; Dweck, 2006). These beliefs have implications for how people respond to feedback about their own performance. A person who believes that her poor performance in a specific domain is an indication that she is WTA on an immutable trait may feel helpless to change the situation. She may be unable to transform feelings of threat stemming from a WTA belief into motivation to take remedial action. In contrast, people who believe that their personal characteristics are malleable are more likely to attribute their negative performance to effort, and are more likely to take remedial action (Hong et al., 1999). Thus, entity and incremental beliefs likely moderate the likelihood of people moving beyond feelings of threat to the subsequent steps necessary for successful behavioral change. Similarly, depression is associated with a perceived lack of control over one’s own outcomes (Garber et al., 1979; Brown and Siegel, 1988). Consequently, people with depressive symptomology who experience feelings of threat may entirely avoid the domain in which they feel WTA rather than turn their attention toward relevant social models who could otherwise help to improve their performance (Abramson et al., 1978; Peterson et al., 1993).

### Social Attention: The Role of Individual Differences in Social Referencing

Social referencing refers to the tendency of a person to look to another person in ambiguous situations to obtain clarifying information. Social referencing behavior appears as early as the first year of life – 10–13 months old infants encountering loud (i.e., potentially exciting but also potentially frightening) mechanical toys will check their caregivers’ facial expressions before touching the toy (Walden and Ogan, 1988). Social referencing is an early-developing component of a set of competencies (which also includes theory of mind, the ability to recognize when information is needed and from whom to seek it, and the ability to signal that information is wanted) that is necessary for developing expertise in social information gathering (Baldwin and Moses, 1996). Individual differences have been observed in social referencing behavior (Dickstein et al., 1984) as well as in related competencies including theory of mind (Cutting and Dunn, 1999; Carlson and Moses, 2001) and social signaling (Walden et al., 1997). Children with Autism Spectrum Disorder, for example, typically show reduced attention to faces and people as well as impairments in social orienting and joint attention (Maestro et al., 2002; Dawson et al., 2004; Chawarska et al., 2010). Cross-cultural data further supports the idea of a “broader autism phenotype” in the general population (Wakabayashi et al., 2006), and evidence suggests that the traits associated with such a phenotype are normally distributed (Hurst et al., 2007). Traits that influence whether an individual will turn his or her attention to social stimuli in an ambiguous or threatening situation could lead to downstream consequences for the ability of an individual to profit from WTA beliefs. It is possible that people with ASD might be less concerned about their relative standing. Yet, to the extent that they are concerned with their social standing or fitting in with their peer group more broadly, due to an inability to seek out adequate social models, this research suggests they should be less likely remedy their social situation.

### Social Approach: The Role of Social Anxiety and Neuroticism

Individual differences in social anxiety and neuroticism both influence the tendency to engage in social approach, particularly during the experience of threat. Social anxiety is characterized by a persistent tendency to avoid social situations involving unfamiliar people or possible scrutiny by others (American Psychiatric Association, 2013). In addition to influencing a person’s chronic or baseline tendency to engage in social approach behavior, social anxiety’s effects seem to be exacerbated by stress. Compared to non-anxious individuals, people with social anxiety disorder react to acute social stress with heightened sensitivity to angry faces and greater social avoidance behavior (Roelofs et al., 2009). These findings suggest that social anxiety might play a critical role in whether an individual engages in social approach (such as seeking feedback) following a threatening WTA belief. Neuroticism, sometimes called emotional instability, is characterized by the tendency to experience negative emotions such as anger, anxiety, and sadness (Costa and MacCrae, 1992). People higher in neuroticism have been found to react to a broad range of stressors with lower levels of problem solving and higher confrontation, avoidance, and self-blame, as well as higher levels of interpersonal withdrawal (O’Brien and DeLongis, 1996; Lee-Baggley et al., 2005). In sum, social anxiety and neuroticism may both be particularly perverse because these traits lead people to withdraw socially and avoid seeking social support at precisely the moments at which such strategies could be the most helpful – such as in the context of a threatening WTA belief.

### Feedback: The Role of Trait Anxiety and Emotional Intelligence

The final step in our proposed conceptual framework that links WTA beliefs to positive long-term changes is the use of social feedback to guide behavioral change. Across many domains, people regulate their performance by monitoring how well they are doing: if they fall short of their desired standard, they change their behavior to try to meet the standard, followed by self-monitoring, in a feedback loop that continues until they are satisfied with their performance (Carver and Scheier, 2000). Trait levels of anxiety may play a critical role at this junction, as research suggests that people are willing to accept both the reasonable and unreasonable advice that they are presented with, after being led to feel anxious (Gino et al., 2012). People who are prone to experiencing anxiety across various situations might benefit less from WTA beliefs because they are less able to distinguish between feedback that is or is not likely to lead to successful behavioral remediation.

Emotional intelligence may also affect a person’s ability to benefit from WTA beliefs. People who score higher on measures of emotional intelligence are better able to predict how they will react to future situations and regulate their emotional experiences to promote goal attainment (Dunn et al., 2007; Mayer et al., 2008; Brackett et al., 2011). Consequently, emotional intelligence may help people effectively regulate their feelings of threat that initially coincide with WTA beliefs, and to skillfully use WTA beliefs to motivate adaptive and approach-oriented future actions. More generally, the example of emotional intelligence highlights the possibility that some of the individual differences we have discussed may have effects at more than one of the critical junctions linking WTA beliefs to long-term benefits. For example, people who score lower on emotional intelligence often have poorer social skills (Frederickson et al., 2012). Thus, people who score lower on measures of emotional intelligence might be less willing or able to seek out advice from relevant social models, or less able to identify appropriate social models.

### The Role of Cultural Context

Culture can influence whether and how individuals initially experience WTA beliefs. In East Asian cultures, self-effacing biases are more common and self-enhancing biases are less common than in Western cultures (Heine and Hamamura, 2007). Because WTA beliefs are also more likely to be the norm in collectivist cultural contexts (Mezulis et al., 2004), WTA beliefs might be less likely to trigger feelings of threat in these contexts or motivate behavioral remediation. Thus, the exact sequence of events proposed in our conceptual framework may also vary across cultures.

## Applying the Conceptual Framework

Next, to demonstrate the relevance of our conceptual framework for understanding when, how, and for whom WTA beliefs can have long-term benefits, we will apply our framework to the domain of skill learning. Although we discuss only one example in depth, similar logic could be applied to extend our conceptual framework to a broad range of psychological domains ranging from friendship formation to political and moral psychology.

### Skill Learning

Our framework suggests that WTA beliefs can have positive long-term benefits for the ability to learn and master new skills. Skill learning meets the pre-conditions of our framework because skill learning is a domain where people are motivated and can improve their own performance through effort. Individuals are motivated to learn and master new skills in part because it feels good to do so: mastery is a powerful predictor of subjective well-being (McGregor and Little, 1998). People are especially motivated to learn and master new skills when they feel like they are not living up to their own expectations (Ryan and Deci, 2000). In work settings, when people’s performance is below their own aspiration levels, they become more likely to search for new strategies and to change their behaviors to try to improve their performance (even if some risk is incurred; Greve, 2003). Furthermore, people work toward learning and developing their skills by observing and seeking critical feedback from others (Henrich and Gil-White, 2001). For these reasons, when people are learning or developing their skills, such as in educational and work settings, WTA beliefs may be especially likely to yield long-term benefits.

Research suggests that WTA beliefs lead individuals to seek out feedback from other people about how to improve their future performance (Walker and Smither, 1999), which can enhance performance on various lab-based tasks (Badami et al., 2012). In one of the few longitudinal studies in this area, managers who initially received the poorest feedback from their coworkers, and who used this feedback to seek out constructive comments from their peers, demonstrated the greatest performance gains over a 5-year period as compared to managers who did not seek out peer feedback (Walker and Smither, 1999). This research provides indirect evidence that WTA rather than BTA beliefs will facilitate the greatest gains in skill learning over time – especially when individuals seek out peer feedback and are provided with the opportunity to practice and develop their skills.

Indeed, the process of feeling WTA, seeking feedback, and using this feedback to improve one’s skills is likely to unfold over time, given that many skills that are relevant to education and employment, such as reading or learning a new computer program, are ongoing processes that take people many years to master. Although some of our theorizing awaits empirical confirmation, research suggests that BTA beliefs may promote idleness and stagnation in one’s skills. For example, success in prior endeavors can paradoxically lead people’s future performance to decline, an effect that is mediated by the complacency that is promoted by overconfidence (Audia et al., 2000). Recent empirical evidence also suggests that overconfidence can have detrimental longitudinal impacts on leadership abilities because overconfident leaders are unable to see their deficiencies and fail to correct for them (Shipman and Mumford, 2011).

#### Skill Learning: Feelings of Threat and the Role of Entity/Incremental Theories

People’s entity/incremental theories are likely to moderate the benefits of WTA beliefs for skill learning. If skills are seen as fixed, then perceiving oneself as WTA may only incur the downsides of anxiety and reduced self-esteem, since no avenue for remediating one’s current skills deficits may appear available. The belief that skills can be grown, on the other hand, may nurture persistence of effort and adaptive change in response to WTA beliefs (Dweck, 1986; Butler, 1987). Across a variety of skills including motor learning (Wulf et al., 2012), exercise efficiency (Stoate et al., 2012), and management abilities (Brown et al., 2016), individuals with a malleable view of their performance show improvements in skill learning compared to individuals with a fixed view of their performance. These gains occur in part because negative feedback does not provide a global threat to self for these individuals, decreasing the need to self-affirm after receiving negative performance feedback, which provides individuals with more time to focus on improving personal performance (Wulf et al., 2012).

In fact, for individuals with a growth mindset, failure can promote learning and superior performance (Mangels et al., 2006). Those with a growth view of their abilities tend to respond to negative performance feedback by searching for new strategies to improve performance (i.e., by examining the strategies of those who outperformed them), whereas individuals with a fixed view of their abilities are more concerned with shoring up their global self-regard after receiving equally negative feedback (i.e., by examining the strategies of those who performed worse than they did; Nussbaum and Dweck, 2008). Thus, in the domain of skill learning, the benefits of WTA beliefs may accrue preferentially to individuals with a growth mindset; for individuals with a fixed mindset, WTA beliefs may cause a loss of interest and disengagement (Bandura and Jourden, 1991), a response that, in many settings, may be even less adaptive than the complacency cultivated by BTA beliefs.

Depression may also preclude individuals from selectively turning their attention toward social models and from seeking out critical skill-relevant feedback following WTA beliefs. People with depression are more likely to give up after experiencing failure on novel tasks and perform more poorly compared to age-matched controls (Holmes and Pizzagalli, 2007). People who report greater depressive symptomology are more likely to seek out more negative feedback from peers after initially receiving performance-relevant negative feedback (Casbon et al., 2005). Thus, depression may lead people to avoid the domain in which they feel WTA, or to look for negative feedback that reinforces their WTA beliefs, rather than to turn their attention toward relevant social models who could facilitate skill-learning.

Self-determination theory (SDT) could be another useful theory to understand when the negative emotions that arise from WTA beliefs translate into adaptive action, such as enhanced skill-learning. SDT predicts that WTA beliefs would be most be most adaptive when people feel autonomous (as if they have control over their behavior in a given domain), competent (like they have skills and are able to improve in a certain domain) and related to others (like they belong or are connected and accepted by others in a certain domain). SDT also illuminates when WTA beliefs are likely to have positive long-term effects for motivation and behavioral remediation. In domains where people are intrinsically motivated, behavior is guided by an internal locus of control, and relevant regulatory processes are based on interest, enjoyment, and personal satisfaction, WTA beliefs are likely to have positive downstream consequences. In contrast, in domains where people are extrinsically motivated, and behavior is guided by an external locus of control, and relevant regulatory processes are driven by rewards and punishments, WTA beliefs might result in reduced motivation or disengagement. Future work should substantiate these theoretically-motivated claims.

#### Social Attention: The Role of Individual Differences in Social Referencing

Individuals who exhibit less spontaneous social attention and less interest in social interactions should also be less likely than other people to seek out relevant social models in response to a WTA belief. there is a great deal of related research suggesting that individuals with Autism Spectrum Disorder (ASD) traits are less likely to attend to other people in their social environment, which can impact skill learning and performance over time. People with ASD are less likely to imitate and attend to the actions of their peers – two behaviors that are critical for communication and skill development (Toth et al., 2006). More specifically, these social attention deficits are associated with difficulties maintaining jobs, despite the fact that the majority of individuals with ASD do not experience cognitive deficits (Nesbitt, 2000). Although more research is needed to explore how ASD traits impact skill learning following WTA beliefs, we speculate that individual differences related to social attention and social interest likely moderate the ability to learn and develop new skills following WTA beliefs, as they prevent individuals from attending to relevant and useful models.

#### Social Approach: The Role of Social Anxiety and Neuroticism

Our framework suggests that people who experience WTA beliefs in combination with social anxiety or neuroticism are less likely to seek out feedback from relevant social models – instead, they might choose to engage in avoidance-related coping strategies. Indeed, people who report higher levels of neuroticism may also be less likely to seek out feedback following WTA beliefs; indeed, individuals who report higher levels of neuroticism report feeling more negative about interacting with another colleague at their workplace whom they believe is performing better than they are (Buunk et al., 2001). Thus, individuals who experience greater social anxiety and neuroticism might be less likely to turn to other successful individuals for skill-related feedback and advice, therefore limiting the ability of WTA beliefs to translate into improved performance and mastery over time.

#### Feedback: The Role of Trait Anxiety and Emotional Intelligence

More general feelings of anxiety may also limit the benefits of WTA beliefs on skill learning, by negatively impacting an individual’s ability to accept and effectively incorporate social feedback into her attempts at behavioral remediation. After receiving critical feedback, individuals with higher self-reported trait anxiety are more likely to feel personally threatened and experience decreases in self-efficacy compared to individuals with lower self-reported trait anxiety (Frey et al., 1986). The decrements in self-efficacy that follow from the receipt of critical feedback are linked to decreased performance, such as lower job-relevant task performance (Randhawa, 2004).

Such performance decrements occur in part because after the receipt of critical feedback socially anxious individuals are less likely to seek out information that might help them to improve their performance. For example, in one study, students were provided with fictitious intelligence feedback that was either negatively or positively discrepant with their self-evaluations (Frey et al., 1986). Students were then provided with the opportunity to read one of several articles that either argued in favor of intelligence testing or derogated intelligence testing. In contrast to students with lower levels of anxiety, who showed no difference in their article choice as a result of the feedback they received about their intelligence, students with higher levels of generalized anxiety were more likely to select articles that criticized intelligence testing after receiving negative information about their intelligence. Although more research is needed to directly illustrate our point that individuals with higher levels of trait anxiety will be less able to make use of WTA feedback to improve their long-term performance, these studies provide suggestive evidence that people who generally experience greater anxiety may be less likely to benefit from WTA beliefs.

In contrast, people who are emotionally intelligent might stand to benefit most from WTA beliefs. Consistent with this possibility, individuals who score higher in emotional intelligence are better able to regulate their emotions in response to experiencing stressful life events, such losing one’s job (Troy et al., 2010). In turn, enhanced emotion regulation can buffer against the negative effects of life stressors on mental health outcomes, such as depression (Robinson et al., 2012). More specific to our conceptual model, people with higher emotional intelligence respond more positively in the face of challenging situations. For example, although engaging in a challenging work experience can sometimes lead employees to feel incompetent, individuals who scored higher in emotional intelligence reported greater feelings of challenge, greater positive affect, and lower intentions to quit their jobs compared to individuals who scored lower in emotional intelligence (Dong et al., 2014). These empirical findings suggest that individuals who score higher in emotional intelligence may be better equipped to transform WTA beliefs into long-term psychological and performance benefits.

In sum, the evidence that we have presented in this section supports the idea that WTA beliefs might incur long-term benefits related to motivating and improving skill learning, and it also identifies gaps in the literature where more work would be necessary to substantiate the claims set by our conceptual framework.

## Discussion

Social comparison is an inescapable aspect of human psychology – as we navigate our social worlds, it is common and natural for us to wonder how we are doing compared to our peers. Although the belief that one is doing better may be comforting in the short-term, the feeling that one is doing worse than one’s peers may have long-term benefits. We have argued that these benefits may have been overlooked by researchers in part because the benefits of WTA beliefs unfold over a longer time scale than can be captured in a typical lab-based study. We have also proposed a conceptual framework to understand when and for whom WTA beliefs are likely to yield long-term benefits. We have proposed that WTA beliefs are most likely to incur long-term benefits when they facilitate adaptive social attention, social approach, incorporation of feedback and behavioral remediation. Finally, we have proposed that WTA beliefs may yield benefits above and beyond other types of negative self-evaluations because the belief that one is performing below the average level of one’s peers is uniquely motivating and promotes socially-focused behavioral remediation. We have applied our conceptual model to explore the potential benefits of WTA beliefs in one specific domain – skill learning. However, empirical research suggests that WTA beliefs occur across diverse domains; thus, the long-term benefits of WTA beliefs should also extend to other domains. As proposed in our conceptual framework, for a WTA belief to incur long-term benefits, an individual must feel threatened and/or motivated to reduce a WTA belief, and an individual must believe that his or her standing in a relevant domain is subject to change. Based on these criteria, another domain whereby WTA beliefs should lead to long-term psychological benefits is friendship formation. The ability to form and maintain friendships is a critical determinant of subjective well-being and physical health (Cacioppo and Patrick, 2008) and forming and establishing social connections is a salient goal for most individuals (Kahneman et al., 2004). Most critically for our model, people can readily influence the quantity and quality of their day-to-day social interactions (Sandstrom and Dunn, 2014). Thus, friendship formation is a domain that meets the pre-conditions of our conceptual framework as a domain where WTA beliefs might potentiate benefits over time.

Indeed, in our own recent research, conducted with nearly 400 first-year university students, participants who believed that they were worse off socially (i.e., had made fewer new friends) than the average first-year student reported lower momentary well-being and belonging (Whillans et al., 2017). Nevertheless, the same students that held WTA beliefs about their social success reported making more close friends 3 months later (controlling for the number of close friends they already had). Thus, our own research – conducted with a large sample of students who were assessed over several months – supports our proposition that WTA beliefs can incur long-term benefits for friendship formation. Additional research is needed to explore the potential moderating conditions proposed by our framework, such as whether individuals who hold entity beliefs about their personality are less likely to reap the social benefits of WTA beliefs (see Howe and Dweck, 2016, for additional discussion).

Another area in which the long-term behavioral and emotional effects of WTA beliefs have been under-investigated is moral standing. A diverse body of social-psychological research has demonstrated that people care deeply about seeing themselves as morally good and that moral self-regard (“Am I a good person?”) responds dynamically to situational cues and feedback from the social environment (Monin and Jordan, 2009). Yet little is known about the long-term impact of believing oneself to be less (or more) virtuous than the average person. In lab studies, participants have been shown to resent and put down “moral rebels” who behave in an ethically superior way (e.g., refusing to complete a racist experimental task) when this implicitly indicts the participants’ own prior behavior (e.g., completing the task; Monin et al., 2008). Similarly, in other studies, people ascribed negative qualities to moral vegetarians, particularly when thinking about the ways that they imagined the vegetarians might judge their own morality, a phenomenon that was dubbed “do-gooder derogation” (Minson and Monin, 2012). These examples suggest that feeling bad about one’s moral standing relative to others – that is, WTA beliefs in the moral domain – may lead to petty takedowns of others, an uncontroversial undesirable consequence. However, participants’ behavioral response options for dealing with their (presumed) feelings of moral inferiority were quite constrained in these paradigms (e.g., they did not have the option of demonstrating their own morality or improving their own moral choices). It is possible that as with receiving more general negative performance feedback, short-term harms may give way to longer-term growth after a person comes to see himself or herself as morally WTA in a particular domain.

Indeed, there is some evidence that even in the short term, feeling less than adequate morally may engender positive behavior change, at least when an avenue to moral self-improvement is made available (Merritt et al., 2010). When people were asked to write about a past misdeed, they were subsequently more likely to express prosocial intentions for their future behavior, apparently as a means of repairing their moral self-regard (Jordan et al., 2011b). Similarly, after being assigned to write about themselves in a negative way, people donated more to charity than they did otherwise (Sachdeva et al., 2009). Exposure to other people’s moral heroism, which could be assumed to make one feel less confident about his or her own moral standing, has also been shown to inspire feelings of elevation and consequent prosocial behavior in some conditions (Schnall and Roper, 2012). Because all of these studies have looked exclusively at immediate consequences in response to situational manipulations in a lab setting, it is unclear how people’s responses to more stable WTA or BTA beliefs about moral standing may affect behavior and emotions as they unfold over time. We argue that at least for people who believe that one’s moral goodness (or badness) is not permanently fixed and can instead be changed effortfully (Chiu et al., 1997), WTA beliefs about moral standing are likely to elicit a long-term process of seeking moral self-improvement.

Our conceptual framework focuses on what happens after WTA beliefs arise, regardless of how they arise. However, there are many interesting and potentially generative points of speculation about how the causes of WTA beliefs and/or the characteristics of WTA beliefs could moderate the long-term benefits, such as how accurate the WTA beliefs are, how far away the WTA beliefs are from the perceived social “average” and the extent to which people hold WTA beliefs across multiple personally-relevant domains. For example, the extent to which people’s WTA beliefs reflect the reality of their own and others’ standing could moderate the long-term benefits. Most people can accurately reflect on whether they have overly positive or overly negative self-perceptions (Bollich et al., 2015). Furthermore, well-adjusted individuals tend to perceive other people’s personalities accurately (Human and Biesanz, 2011). Research also suggests that the best performing individuals are most likely to be accurately self-aware regarding their own performance (Kruger and Dunning, 1999). This provides empirical evidence that having perceptions about oneself and others that are based in reality is psychologically adaptive. Similarly, WTA beliefs might be most likely to trigger an adaptive cascade of social and behavioral consequences when WTA beliefs accurately reflect reality.

Research suggests that there are three primary causes of overconfidence: people overestimate their abilities, people over-place themselves in relation to others, and people are overconfident in their estimates (Larrick et al., 2007). When it comes to task performance, people often over-place themselves because they are not provided with sufficient incentives to accurately answer, and so accurate placement competes with other motivations such as appearing competent, self-confidence, and modest (Benoît et al., 2015). Following from these studies, WTA beliefs might therefore sometimes arise in domains whereby underestimating one’s placement is more socially desirable than overestimating one’s placement. Although our model does not specifically address how the cause of WTA beliefs predict behavioral remediation, it is possible that WTA beliefs might be more likely to inspire action when they are not accompanied with underconfidence in one’s ability – as this could make people less likely to change their behavior or more likely to self-handicap. Future work should further substantiate this claim. Because our theoretical model is agnostic about the origins of the WTA beliefs, additional research is needed to disentangle whether and how the characteristics of the original WTA beliefs themselves – including whether or not these beliefs are accurate – shape the downstream motivational and behavioral consequences.

The distance of an individual’s WTA belief from a perceived social average could also moderate the long-term benefits. For example, individuals who believe that they are further from a relevant social “average” might be less likely to believe that they can successfully remedy their own behavior and may be discouraged by a WTA belief. Having a WTA belief that is distant enough from the perceived average to be motivating, but also not so distant that it becomes discouraging, might be the most adaptive for promoting the long-term benefits. Although more research is needed to substantiate this claim, this proposition is consistent with classic research in psychology showing that the most adaptive relationship between a set of variables and an outcome is often non-monotonic (e.g., Yerkes and Dodson, 1908).

Relatedly, WTA beliefs might be most beneficial when they occur in one specific domain (versus across multiple domains). This proposition is consistent with theorizing suggesting that positive psychological traits and virtues, such as courage, justice, and optimism, may have negative consequences when experienced too seldom or too frequently (Grant and Schwartz, 2011). For example, holding WTA beliefs across multiple personally relevant domains at the same time is likely to result in a reduced desire or belief in one’s ability to change one’s own performance. More specifically, individuals who feel WTA across multiple personally-important domains, may experience shame that could prevent them from asking for relevant advice (Tracy and Robins, 2006).

Finally, our conceptual model proposes that individuals, after experiencing a WTA belief, should continue to persist in improving their performance by seeking out relevant social models and social feedback, and by using this feedback to encourage behavioral remediation. However, recent empirical and theoretical research suggests that goal disengagement is a fundamental component of effective self-regulation (see Wrosch et al., 2013 for a review). Consequently, in some cases, the most adaptive response to the experience of a WTA belief could be to select another activity with a greater likelihood of improvement. Following from this proposition, another potentially productive area of research is to examine the boundary conditions for when WTA beliefs can be effectively remedied through subsequent approach-oriented actions such as seeking feedback, versus when they would be more effectively remedied through subsequent avoidance-oriented actions such as task disengagement. Future research should examine the specific components of WTA beliefs that predict whether the long-term benefits will arise – such as accuracy, perceived distance from the average, and the specificity of the WTA belief – to better understand whether an individual is likely to proceed through the steps of our proposed framework.

In the current paper, we focused on the extent to which WTA beliefs can result in social attention, feedback, and subsequent improvement in social domains, such as skill-learning and friendship formation. We focused on socially-relevant approach behaviors due to the fact that such solutions are most likely to potentiate long-term benefits. We also focused on socially-relevant approach behaviors due to the fact that past research in this area has typically focused on social outcomes – such as subsequent friendship formation and time spent soliciting input and feedback from peers and colleagues. However, it is possible that people might respond to WTA beliefs using non-social means of improvement such as self-study. People might be most likely to engage in non-social remediation when WTA beliefs elicit defensive or inhibition-based responses that could lead people to distance themselves socially from others. While our current focus on social remediation was chosen based on the focus of past research, a generative area of future research would be to explore the conditions by which WTA beliefs lead to social vs. non-social remediation.

By outlining a conceptual model and proposing when and for whom the benefits of WTA beliefs are likely to arise, this paper speaks to the critical importance of examining the potential of WTA beliefs to provide a springboard to long-term psychological flourishing. It is our hope that this paper will encourage researchers to question not only why WTA beliefs occur, but when WTA beliefs may play an important role in successfully navigating our social environments.

## Author Contributions

AW, AJ, and FC developed the conceptual framework. AW and FC drafted the manuscript. AJ provided critical revisions.

## Conflict of Interest

The authors declare that the research was conducted in the absence of any commercial or financial relationships that could be construed as a potential conflict of interest.

## Footnotes

1 We are not proposing that WTA beliefs preclude the use of non-social methods of improvement, such as spending time alone practicing a novel skill. Instead, we are proposing that in the case of feeling WTA, engaging in socially oriented affective, cognitive, and behavioral changes might be the most direct route to ameliorate the threat elicited by WTA beliefs and to potentiate long-term benefits.
